# Expression Profile of New Gene Markers and Signaling Pathways Involved in Immunological Processes in Human Cumulus-Oophorus Cells

**DOI:** 10.3390/genes12091369

**Published:** 2021-08-31

**Authors:** Błażej Chermuła, Greg Hutchings, Wiesława Kranc, Małgorzata Józkowiak, Karol Jopek, Bogusława Stelmach, Paul Mozdziak, Leszek Pawelczyk, Hanna Piotrowska-Kempisty, Robert Z. Spaczyński, Bartosz Kempisty

**Affiliations:** 1 Division of Infertility and Reproductive Endocrinology, Department of Gynecology, Obstetrics and Gynecological Oncology, Poznan University of Medical Sciences, 33 Polna St., 60-535 Poznan, Poland; blazej.chermula@wp.pl (B.C.); b_stelmach@wp.pl (B.S.); pawelczyk.leszek@ump.edu.pl (L.P.); robert.spaczynski@ump.edu.pl (R.Z.S.); 2 The School of Medicine, Medical Sciences and Nutrition, Aberdeen University, Aberdeen AB25 2ZD, UK; g.hutchings.16@abnd.ac.uk; 3 Department of Anatomy, Poznan University of Medical Sciences, 6 Swiecickiego St., 60-781 Poznan, Poland; wkranc@ump.edu.pl; 4 Department of Toxicology, Poznan University of Medical Sciences, 30 Dojazd St., 60-631 Poznan, Poland; malgorzata.jozkowiak@gmail.com (M.J.); hpiotrow@ump.edu.pl (H.P.-K.); 5 Department of Histology and Embryology, Poznan University of Medical Sciences, 6 Swiecickiego St., 60-781 Poznan, Poland; kjopek@ump.edu.pl; 6 Physiology Graduate Program, North Carolina State University, Raleigh, NC 27695, USA; pemozdzi@ncsu.edu; 7 Prestage Department of Poultry Science, North Carolina State University, Raleigh, NC 27695, USA; 8 Department of Basic and Preclinical Sciences, Institute of Veterinary Medicine, Nicolaus Copernicus University in Torun, 1 Lwowska St., 87-100 Torun, Poland; 9 Department of Veterinary Surgery, Institute of Veterinary Medicine, Nicolaus Copernicus University in Torun, 1 Lwowska St., 87-100 Torun, Poland

**Keywords:** cumulus cells, cytokine action, IVF

## Abstract

The function of the immune system extends from defense against external pathogens to the recognition and elimination of mutated or dying cells, aiding elimination of malignant potential and/or maintaining homeostasis. The many cell types of the immune system secrete a broad range of factors to enable cellular signaling that is vital to physiological processes. Additionally, in the ovary, follicular selection and maturation, as well as ovulation, are directly regulated by the nearby immune cells. Additionally, ovulation and rupture of the follicle have been observed to resemble a local inflammatory response. Cells of the cumulus–oocyte complex (COC) show evolving gene expression profiles throughout the oocytes’ lifespan, including genes associated with immunological processes. Analysis of these genes allows the identification of useful molecular markers, as well as highlighting gene functions and interactions in these cells. Cumulus cells were obtained from hormonally stimulated patients undergoing an in vitro fertilization procedure and studied under long-term culture conditions. The microarray technique made it possible to compare the level of CCs’ gene expression on the 1st, 7th, 15th and 30th day of cultivation. Additionally, RNA microarray analysis was performed to map gene expression in these cells, associated with immunological processes and associated cytokine signaling. Subsequently, the use of DAVID software allowed us to identify the “defense response to other organism”, “defense response”, “defense response to virus”, “cytokine secretion”, “cytokine production” and “cytokine-mediated signaling pathway” GO BP terms, as well as allowing further analysis of the most differentially expressed genes associated with these processes. Of the 122 genes involved, 121 were upregulated and only one was downregulated. The seven most upregulated genes related to the abovementioned terms were ANXA3, IFIT1, HLA-DPA1, MX1, KRT8, HLA-DRA and KRT18. Therefore, genes involved in immunological defense processes are upregulated in CC cultures and could serve as useful molecular markers of growth and development in the COC, as well as the proliferation of granulosa and cumulus cells.

## 1. Introduction

At the pre-antral to antral follicle transition, granulosa cells surrounding the oocyte differentiate into mural cells and cumulus cells (CCs), which are physically separated by the formation of the antrum. CCs then closely associate with the oocyte, forming the cumulus–oocyte complex (COC) and accompany it following release. Successful ovulation and the development of a competent oocyte are dependent on support by the surrounding CCs, which play a role in the metabolism of pyruvate and glucose in the oocyte and continue to proliferate during cumulus expansion, a process occurring just before ovulation [1]. Similar to the oocyte, which releases growth factors influencing the activity in neighboring cumulus cells, the CCs themselves produce cytokines and factors released during ovulation. Bi-directional signaling between CCs and the oocyte in the COC is vital and takes place both through gap junctions (GJs) and paracrine factors. Apart from facilitating the exchange of cytokines and small molecules, GJs also have a role in the regulation of programmed cell death. By arresting meiosis in the oocyte, CCs have an important influence on the development and viability of the oocyte [2,3].

In addition to maintaining homeostasis through recognition and removal of dangerous pathogens in the human body, the immune system and the related cells are well documented to play an important role in ovarian development, follicular selection and ovulation [4]. Additionally, ovarian germline stem cells are regulated by immune cells and their associated factors [5]. Consequently, molecular markers of oocyte lifespan associated with immunological processes have attracted significant scientific interest. Advancing understanding of the molecular interactions involving the oocyte microenvironment throughout the menstrual cycle may have a direct impact on further research in assisted reproductive technologies and ovarian cancer.

Ovulation is characterized by an increase in vasodilation, prostaglandin synthesis, cell proliferation and tissue remodeling in the ovary. These are also hallmarks of an inflammatory response, with the two processes sharing a large number of molecular effector molecules. In total, 259 genes involved in inflammation and innate immunity were previously shown to be significantly upregulated in human granulosa cells (GCs) during ovulation in an in silico study [6].

It is important to identify the most differentially expressed genes related to cytokine action and metabolism-mediated signaling pathway activity, which are involved in immunological defense processes, throughout the lifespan of CC in in vitro culture. Analysis of the most differentially expressed genes provides an insight into the understanding of gene function and interactions in these cells, as well as allowing identification of new cellular markers involved in the abovementioned processes. Although genes involved in immunological processes are significantly upregulated in the cumulus cells’ in vitro lifespan, the exact function and interaction of these genes in vivo remains to be investigated.

In this study, cumulus cells were harvested from an in vitro culture at 1, 7, 15 and 30 days. After RNA isolation, transcriptomic analysis was performed on 22,480 transcripts using the microarray method. Genes whose expression was analyzed over four time intervals were uploaded to the DAVID software platform. During analysis of the microarray results, the main attention was paid to the six ontology groups: namely the “defense response to other organism”, “defense response”, “defense response to virus”, “cytokine secretion”, “cytokine production” and “cytokine-mediated signaling pathway” GO BP terms.

The effectiveness of oocyte IVM procedures in ART is at an unsatisfactory level. Nevertheless, the use of this method is gaining new recognition [7,8]. Most research groups are focusing on developing oocyte in vitro maturation under laboratory conditions. The main goal of other researchers is only to obtain mature oocytes, and other groups do not study the changes that take place in cumulus cells. It seems interesting to learn more about the cells’ changes under completely new conditions, completely different from the physiological ones observed in the ovarian follicle. Despite many attempts and efforts, it is difficult to recreate conditions even slightly similar to those observed in the ovarian follicle.

The focus of the study was to evaluate the expression of genes involved in immunological processes, derived from patients treated in an IVF program. Long-term CC in vitro cultivation was performed. The 30-day in vitro culture of CCs revealed the directions in which these cells can differentiate under laboratory conditions.

## 2. Materials and Methods

### 2.1. Cumulus Cell Collection

For our studies, CCs from 12 infertile female patients undergoing controlled ovarian hyperstimulation during an in vitro fertilization procedure were used. For this research, patients between 25 and 40 years old were qualified after giving their full written consent. The IVF-ICSI procedure was performed in Centre of Diagnosis and Treatment of Infertility at the Division of Infertility and Reproductive Endocrinology, Poznan University of Medical Sciences. For this study, patients classified as poor ovarian responders, with an antimullerian hormone (AMH) serum level less than 0.7 ng/mL, were excluded. Additional exclusion criteria were: previously diagnosed endometriosis, polycystic ovary syndrome (PCOS), less than 9 antral follicles observed during patient ovary ultrasonography and follicle-stimulating hormone (FSH) serum levels on Days 2–3 higher than 15 mU/mL. During controlled ovarian hyperstimulation, patients were administered highly purified human menopausal gonadotropin (hMG; Menopur, Ferring Pharmaceuticals Poland sp. z o.o., Poland) and recombinant human follicle-stimulating hormone (rhFSH; Gonal F, Merck sp. z o.o., Poland or Puregon, MSD Poland sp. z o.o., Poland). In addition, to stop pituitary activity, patients received Orgalutran in an antagonist protocol (0.25 mg ganirelix, MSD Poland sp. z o.o., Poland) or Cetrotide (0.25 mg cetrorelix, Merck sp. z o.o., Poland) injections. To initiate ovulation, patients were administered an injection of rhchorionic gonadotropin (rhCG; Ovitrelle, 250 μg, Merck sp. z o.o., Poland). Collection of oocytes was performed by transvaginal ultrasound examination 36 h after rhCG administration.

After the oocyte pick-up (OPU) procedure, cumulus–oocyte complexes (COCs) were selected for oocyte denudation and further ICSI fertilization. Denudation is a routine procedure of oocyte preparation before intracytoplasmic sperm injection. This process included an enzymatic (HYASE-10X is 800 IU/mL) and mechanical removal of several layers of somatic cumulus cells which adhered to the oocyte’s ZP (zona pellucida). In our studies, we recovered 10 COCs on average from each patient (mean: 10 COCs ± 3.38 SEM (standard error of the mean); range 5–15 COCs). For further study, cumulus cells obtained in this way from each patient were pooled together. The Poznan University of Medical Sciences Bioethical Committee approved this research through Resolution No. 1290/18.

### 2.2. Primary Long-Term In Vitro Culture of Cumulus Cells

CCs obtained during the denudation process were washed twice through centrifugation at 200× *g* for 10 min at room temperature (RT) in a culture medium consisting of Dulbecco’s Modified Eagle Medium (DMEM, Sigma; Merck KGaA, Darmstadt, Germany), 10,000 µg/mL streptomycin and 10,000 U/mL penicillin (Invitrogen; Thermo Fisher Scientific Inc., Waltham, MA, USA), 10 mg/mL gentamicin (Invitrogen; Thermo Fisher Scientific Inc., Waltham, MA, USA), 2% fetal bovine serum (FBS, Sigma; Merck KGaA, Darmstadt, Germany) and 4 mM L-glutamine (stock 200 mM, Invitrogen; Thermo Fisher Scientific Inc., Waltham, MA, USA). After determining the number of cells using the “Neubauer improved” counting chamber (ISO LAB Laborgerate GmbH, DIN Certificate EN ISO 9001), samples with more than 90% cell viability were used for further long-term (30 days) primary in vitro culture. In this study, four periods of culture (24 h, and the 7th, 15th and 30th days) were investigated. CCs were cultured at 37 °C and 5% CO_2_. After reaching 90% confluence, the cells were detached from the culture plate through treatment with 0.05% trypsin-EDTA (Invitrogen; Thermo Fisher Scientific Inc., Waltham, MA, USA) for 1–2 min and counted by using an ADAM Cell Counter and Viability Analyzer (ADAM CCVA) (Bulldog Bio, Portsmouth, NH, USA) (Adam CCVA). CCs were cultured for 30 days. Cells from each patient were cultured individually. Each culture week, the culture medium was changed twice. At 24 h, and 7, 15 and 30 days of culture, total RNA isolation was performed [9].

The primary in vitro culture was carried out for 30 days divided into 4 time intervals. The first was the 0 point (24 h), which revealed the physiological properties of the cells observed in vivo. The following days showed the changes in the cultures: the 7th day of in vitro culture determined the short-term culture, while the 15th day showed the effects of the first passage. On Day 30, it was possible to note the changes that occurred at the end of long-term in vitro culture.

### 2.3. Total RNA Extraction

Using the Chomczyński–Sacchi method [10], total RNA was isolated from the CCs of each of the 12 patients after 24 h, and 7, 15 and 30 days of culture. According to the isolation protocol, CCs were suspended in 1 mL of a phenol and guanidine thiocyanate monophase solution (TRI Reagent, Sigma-Aldrich, St. Luis, MO, USA). Next, chloroform was added to this mixture. This resulted in the appearance of three phases, with RNA located in the uppermost aqueous phase. In the next step, to the RNA pellet, Phase 2-propanol (Sigma; Merck KGaA St. Luis, MO, USA; catalog number I9516) was added and the pellet was washed with 75% ethanol. RNA extracted in this way was resuspended in 10 μL of pure water and used for further analysis. To assess the total RNA amount and purity, a NanoDrop spectrophotometer (Thermo Scientific, Warsaw, Poland) was used. The RNA amount was determined through optical density measurements at 260 nm. Total RNA purity was estimated by using the 260/280 nm absorption ratio. Samples from 12 patients with an 260/280 nm absorbance ratio greater than 1.8 were used for RT-qPCR analysis [11,12]. For microarray expression, the 12 trials obtained were randomly divided into 2 equal groups which contained RNA from 8 patients.

### 2.4. Microarray Expression Analysis

Total RNA (100 ng) was converted to double-stranded cDNA. In the next step, labeled complementary RNA (cRNA) was synthesized and amplified in vitro through transcription of the double-stranded cDNA template (GeneChipTM 3′IVT PLUS Reagent Kit, Applied Biosystems, Foster City, CA, USA). The obtained cRNA was fragmented by divalent cations and elevated temperature. Fragmented and labeled cRNA (7.5 μg) was hybridized to the Human Genome U219 Array Strip (45 °C/16 h, Applied Biosystems, Foster City, CA, USA). Next, the microarrays were washed and stained according to the technical protocol using the Affymetrix GeneAtlas Fluidics Station. Subsequently, the array strips were scanned using the Imaging Station of the GeneAtlas System. The preliminary analysis of the scanned chips was performed using the Affymetrix GeneAtlas operating software. The quality of the gene expression data was checked according to the quality control criteria provided by the software. The CEL files obtained were imported into downstream data analysis software. All of the analyses and graphs presented here were performed using the Bioconductor and R programming languages. Each CEL file was merged with a description file. In order to correct the background and normalize and summarize the results, we used the Robust Multiarray Averaging (RMA) algorithm. 

Statistical significance of the analyzed genes was evaluated using moderated *t*-statistics via the empirical Bayes method. The obtained *p*-value was corrected for multiple comparisons using Benjamini and Hochberg’s false discovery rate. The selection of significantly changed gene expression was based on a *p*-value below 0.05 and an expression fold higher than 2. Differentially expressed genes were subjected to the selection of genes involved in cytokine action and metabolism-mediated signaling pathway activity during immune defense processes in human cumulus-oophorus cells (CCs). Differentially expressed gene lists were uploaded to the DAVID software (Database for Annotation, Visualization and Integrated Discovery), where “defense response to other organism”, “defense response”, “defense response to virus”, “cytokine secretion”, “cytokine production” and “cytokine-mediated signaling pathway” GO BP terms were obtained. Expression data of these genes was subjected to hierarchical clusterization procedure and presented as a heatmap graph. Detailed analyses of genes belonging to selected GO BP terms were presented as plots using the “GOplot” library [13]. 

Moreover, a list of differentially expressed genes from selected GO BP terms was uploaded to the STRING software (Search Tool for Retrieval of Interacting Genes/Proteins) for interaction prediction.

The ReactomeFIViz app that connects with the Cytoscape software for creating the Reactome Functional Interaction (FI) network was employed to identify the set of differentially expressed genes.

Finally, ene expression data was mapped on pathway graph based on KEGG (Kyoto Encyclopedia of Genes and Genomes) database using the “pathview” library [14].

### 2.5. RT-qPCR Analysis

The RT-qPCR (Reverse Transcription quantitative PCR) analysis was performed to verify the microarray results. Six most significantly upregulated genes were selected from the GOs of interest. Using three biological samples for each time period, the changes in their expression were analyzed. Reverse transcription was performed using the Transcriptor First Strand cDNA Synthesis Kit (Roche Diagnostic GmbH, Mannheim, Germany) and a Mastercycler nexus gradient (Eppendorf, Hamburg, Germany) thermocycler. For the reverse transcription, 200 ng of RNA was used. 

The qPCR was performed using the Light Cycler^®^ 96 (Roche Diagnostic GmbH, Germany), RT2 SYBR^®^ Green ROX™ qPCR Master Mix (Qiagen Sciences, Inc., Venlo, The Netherlands), and sequence-specific primers (Table 1). 3-phosphate glyceraldehyde de-hydrogenase (GADPH), hypoxanthine 1 (HPRT1) phosphoribosyltransferase and β-actin (ACTB) were used as housekeeping genes. The gene expression was analyzed using relative quantification (RQ). The RT-qPCR primers were designed by using Primer3Plus software (primer3plus.com/cgi-bin/dev/primer3plus.cgi). Reaction was performed according to the thermocycling protocol: preincubation at 37˚C (30 s); 3 step amplification (95 °C—15 s, 58 °C—15 s, 72 °C—15 s) for 45 cycles; melting (95 °C—60 s, 40 °C—60 s, 70 °C—1 s, 95 °C—1 s); cooling at 37 °C (30 s). The RT-qPCR analysis was conducted in relation to the 24 h of in vitro culture. All of the changes presented on the graph were statistically significant (between time periods) at *p* < 0.05. For gene expression analysis, the 2 ΔΔCq method was used [15].

### 2.6. Statistical Analysis

Statistical analysis of our results was conducted using the Bioconductor software (v. 3.1.0,) and R 3.5.1 programming language. To determine statistical significance of the analyzed genes, the empirical Bayes method was used. To correct the results for multiple comparisons, Benjamini and Hochberg’s false discovery rate was employed (*p* < 0.05 indicated a statistically significant difference). Statistical significance estimation of the enriched GO terms and Kyoto Encyclopedia of Genes and Genomes (KEGG) pathways was based on the DAVID database software (v.6.8, Leidos Biomedical Research, Inc., National Laboratory for Cancer Research, Frederick, MD, USA). Each GO terms and KEGG pathway was considered significantly enriched if it contained at least 5 different expressed genes with *p* < 0.05. Finally, RT-qPCR statistical analysis was performed by using the Real Statistics Resource Pack for MS Excel 2016 (Microsoft Corporation, Redmond, WA, USA) [16,17].

## 3. Results

**Figure 1:**
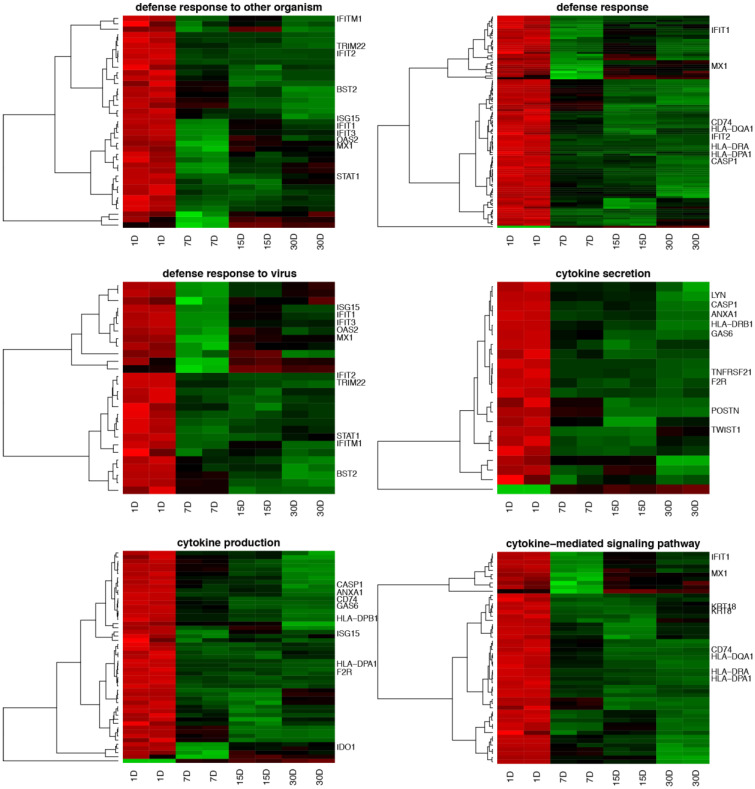
Heatmaps presenting differentially expressed genes involved in “defense response to other organism”, “defense response”, “defense response to virus”, “cytokine secretion”, “cytokine production” and “cytokine-mediated signaling pathway”, based on GO BP terms. Each row on the *Y* axis represents a single transcript. The *X* axis presents the names of the samples, which are the same as the culture time point. The red color indicates downregulated and the green indicates upregulated genes at subsequent points of the analysis. The gene labels are limited to the 10 most changed in a given GO BP process.

The Human Genome U219 Array Strip was used for the microarray gene expression analysis of human cumulus-oophorus cells (CCs), allowing for the study of gene expression of 22,480 transcripts at 1, 7, 15 and 30 days of in vitro culture. Genes with more than a twofold change and corrected *p*-values less than 0.05 were directed for the further downstream analysis. In total, 4773 differentially expressed genes (DEGs) were identified according to the above criteria. The list of DEGs was uploaded to the DAVID software platform, which allowed us to assign the genes to 775 GO BP, 33 GO MF and 125 GO CC gene ontology terms. The focus of the present analysis was placed on genes involved in cytokine action and metabolism-mediated signaling pathway activity during immune defense processes in human cumulus-oophorus cells (CCs). DAVID software indicated the following GO BP terms covering the above processes: “defense response to other organism”, “defense response”, “defense response to virus”, “cytokine secretion”, “cytokine production” and “cytokine-mediated signaling pathway”. In total, 122 genes involved in these processes were hierarchically clustered and presented as heatmaps (Figure 1); 121 genes were upregulated and only one gene was downregulated. In this research, the authors focused on the most upregulated genes. The direction of expression change was maintained in the human cumulus-oophorus cell culture at subsequent points of the analysis (after 7, 15 and 30 days of in vitro culture). The seven most significantly upregulated genes, with their symbols, fold changes and corrected *p*-values, are shown in Table 2. 

**Figure 2:**
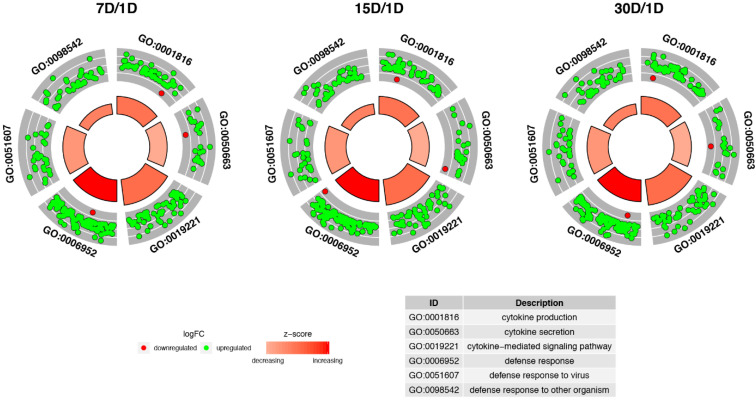
Circular scatterplots of the differentially expressed genes involved in “defense response to other organism”, “defense response”, “defense response to virus”, “cytokine secretion”, “cytokine production” and “cytokine-mediated signaling pathway” GO BP terms. Each dot represents a single gene. The *z*-scores are presented as segments of the inner circles.

The z-scores are important parameters for consideration, as they reveal whether the function represented by the given GO term is more likely to be decreased (negative values) or increased (positive values). The *z*-scores are presented as segments of the inner circles in Figure 2. The expression of most genes was increased (green dots) in all ontological groups. The *z*-scores of the GO BP terms had positive values, indicating that the processes described by these GO BP terms were upregulated. The expression pattern did not change at any of the analyzed time points. Considering the points above, the subsequent analysis was based only on the Day 7–Day 1 comparison.

**Figure 3:**
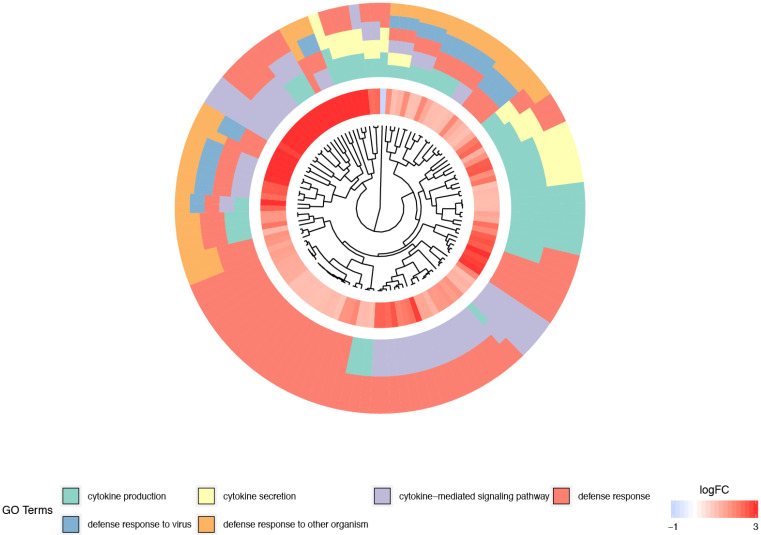
Dendrogram of the differentially expressed genes involved in “defense response to other organism”, “defense response”, “defense response to virus”, “cytokine secretion”, “cytokine production” and “cytokine-mediated signaling pathway” GO BP terms. The DEGs were clustered based on their logFC values.

One of the most visually appealing ways of presenting interactions between genes and GOs is a dendrogram (Figure 3). Clusters contain functionally related genes based on their expression pattern. The middle circle represents the logarithm of fold change (logFC) of the differentially expressed genes assigned to the studied GO terms. The GO terms are shown as the outer ring. Clusters of the same color over the entire width of the outer circle represent genes that are unique for a specific GO term. Clusters of different colors on the cross-section of the outer circle show sets of genes which are likely to be functionally related. 

**Figure 4:**
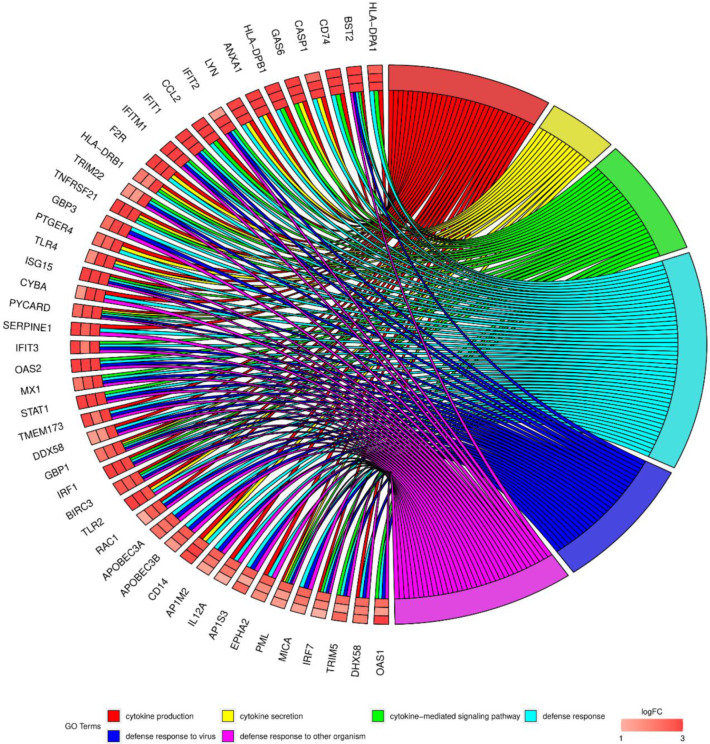
Analysis of enriched Gene Ontology groups involved in cytokine action and metabolism-mediated signaling pathway activity during immunological defense processes in human cumulus-oophorus cells (CCs). The network plot presents the linkages of genes and GO BP terms.

**Figure 5:**
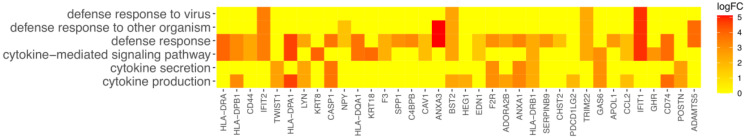
Heatmap presenting the relationship between genes and selected GO BP terms. The yellow tiles indicate the absence of logFC values.

In the Gene Ontology database, single genes may belong to many ontological terms, necessitating the visualization of logFC values (Figure 4) and heatmaps (Figure 5). The strongest upregulated genes from the examined GO BP terms included, among others: ANXA3 (annexin A3), IFIT1 (interferon-induced protein with tetratricopeptide repeats 1), HLA-DPA1 (major histocompatibility complex Class II, DP alpha 1), MX1 (myxovirus (influenza virus) resistance 1, interferon-inducible protein p78 (mouse)), KRT8 (keratin 8), HLA-DRA (major histocompatibility complex Class II, DR alpha) and KRT18 (keratin 18).

**Figure 6:**
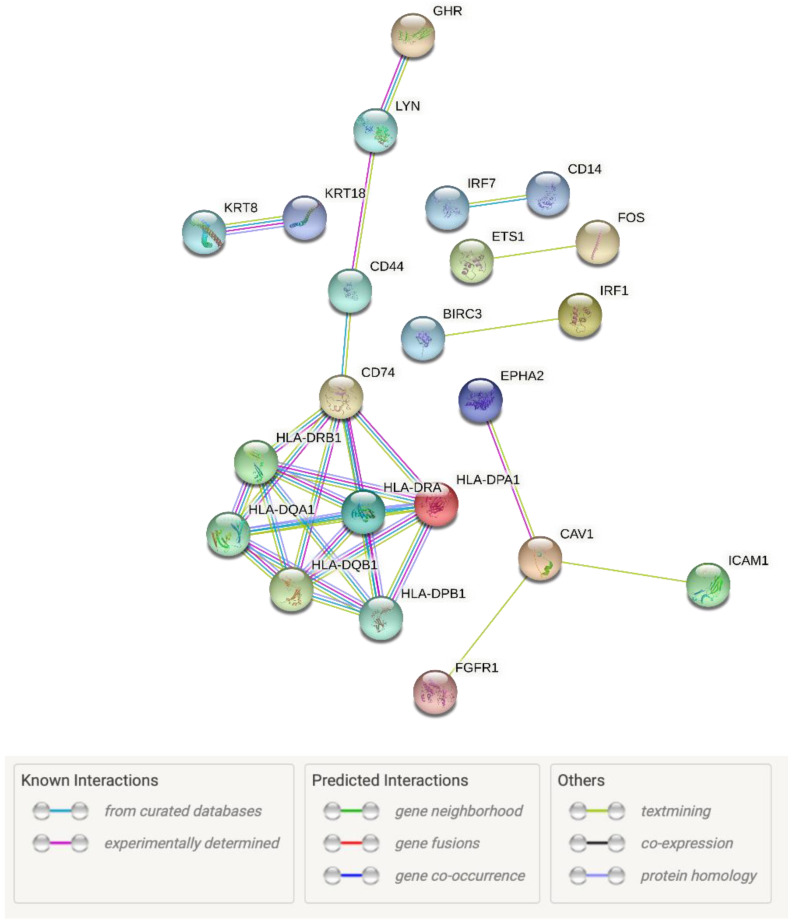
Interaction network of proteins encoded by the 22 most changed DEGs belonging to “defense response to other organism”, “defense response”, “defense response to virus”, “cytokine secretion”, “cytokine production” and “cytokine-mediated signaling pathway” GO BP terms. The network was generated using STRING software. The network nodes represent the structures of proteins encoded by the genes of interest. Empty nodes indicate proteins with an unknown 3D structure.

STRING software was used for predicting the interactions between the proteins encoded by the DEGs belonging to the GO BP terms. The number of genes used to create the STRING interaction network was limited to the 50 most changed DEGs for ease of readability (Figure 6).

**Figure 7:**
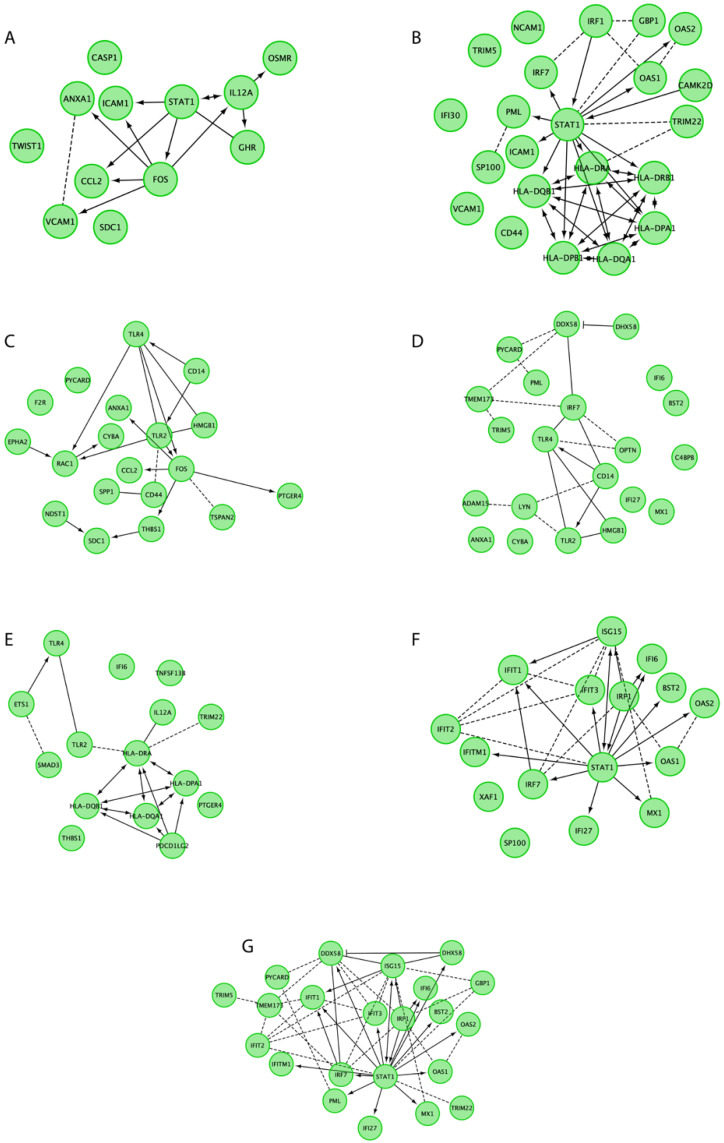
Reactome FI networks between differentially expressed genes belonging to the “defense response to other organism”, “defense response”, “defense response to virus”, “cytokine secretion”, “cytokine production” and “cytokine-mediated signaling pathway” GO BP terms. “--->”, activating/catalyzing; “-|”, inhibition; “-“, FIs extracted from complexes or inputs; “---”, predicted FIs. (**A**) Reactome FI network for the cytokine-mediated signaling pathway. (**B**) Reactome FI network for the interferon-gamma-mediated signaling pathway. (**C**) Reactome FI network for the inflammatory response. (**D**) Reactome FI network for the innate immune response. (**E**) Reactome FI network for the immune response. (**F**) Reactome FI network for the Type I interferon signaling pathway. (**G**) Reactome FI network for the defense response to virus.

In the next part of the analysis, we used the ReactomeFIViz app for an investigation of the functional interactions among the proteins encoded by DEGs belonging to selected GO BP terms (Figure 7).

**Figure 8:**
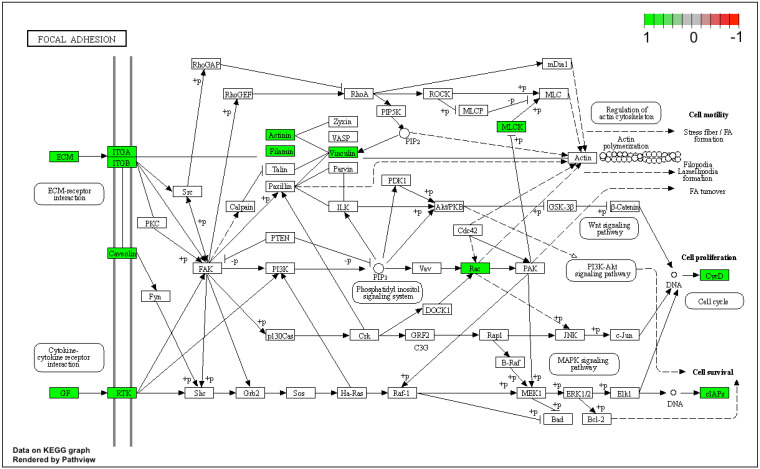
Pathway graph of the focal adhesion signaling pathway based on the KEGG database. The green color indicates parts of the pathway that are upregulated after 7 days of human cumulus-oophorus cell culture in vitro.

**Figure 9:**
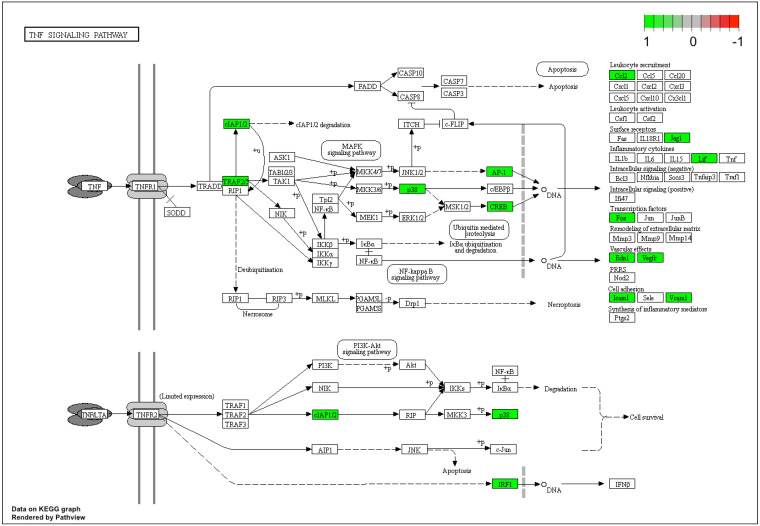
Pathway graph of the TNF signaling pathway, based on the KEGG database. The green color indicates parts of the pathway that are upregulated after 7 days of human cumulus-oophorus cell culture in vitro.

Lastly, gene expression data were mapped based on the “focal adhesion signaling pathway” (Figure 8) and “TNF signaling pathway” (Figure 9) pathway graphs, based on the KEGG (Kyoto Encyclopedia of Genes and Genomes) database. 

**Figure 10:**
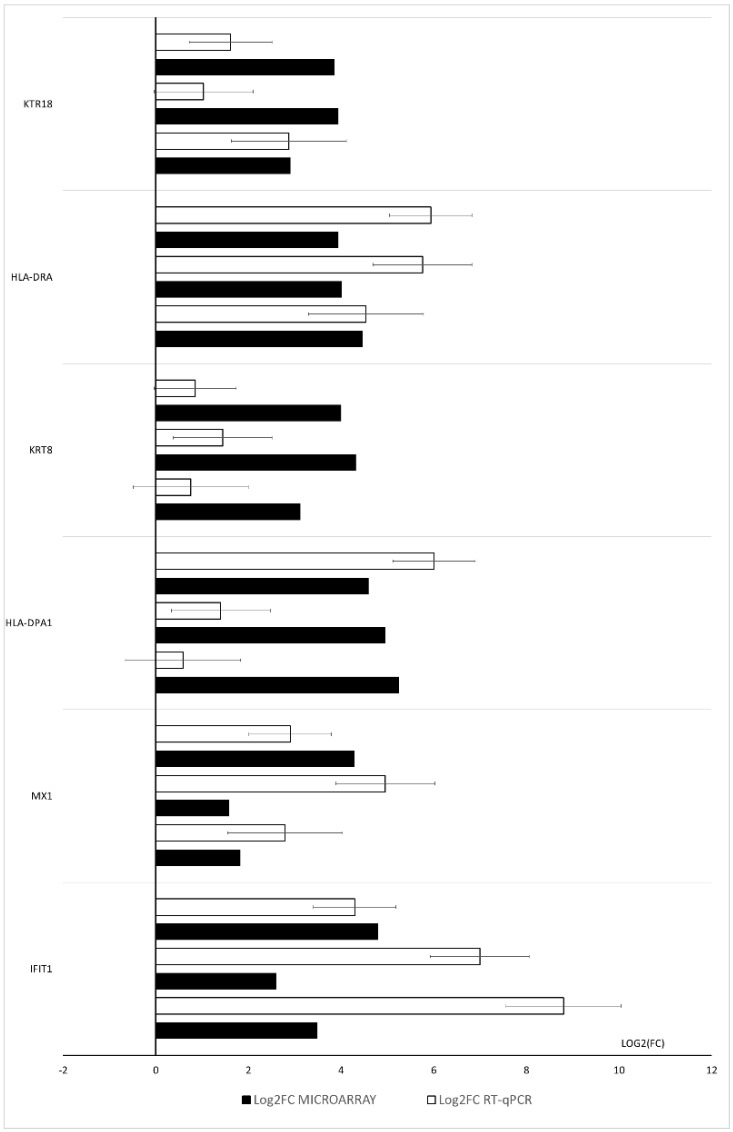
RT-qPCR results. Validation of the microarray results of the six most upregulated genes.

The expression of the six upregulated genes (IFIT1, HLA-DPA1, MX1, KRT8, KRT18 and HLA-DRA) with the highest expression change was validated. The main purpose of the validation was to confirm the direction of expression of the selected genes in relation to the results from the expression microarrays. The validation of results is presented in Figure 10. 

## 4. Discussion

Transcriptomic analysis of human CCs cultured in vitro allowed the identification of genes involved in immunological defense and possible cell markers of the COC’s lifespan, as well as an investigation of the functional interactions among the gene products. Of the differentially expressed genes belonging to the ontological groups “defense response to other organism”, “defense response”, “defense response to virus”, “cytokine secretion”, “cytokine production” and “cytokine-mediated signaling pathway”, the seven most upregulated (ANXA3, IFIT1, HLA-DPA1, MX1, KRT8, KRT18 and HLA-DRA) were further considered for analysis (Table 2). There is little information about pro-inflammatory gene expression analysis in follicular cells. Follicular fluid analysis allowed us to correlate the level of expression of these genes with the quality of the oocytes and pregnancy rates. The current research focused on the analysis of pro-inflammatory genes, and the effect of their on the ovulation process and the quality of oocytes itself [18,19,20].

The first of the analyzed genes belongs to the annexin family of intracellular calcium-dependent phospholipid membrane binding proteins. Annexin A3 (ANXA3) was the most upregulated gene in this study, by 34.27-fold. It encodes an angiogenic factor believed to induce VEGF secretion through the HIF-1 pathway in human umbilical vein endothelial cells [21]. It is also of interest in cancer treatments, as it shows abnormal expression over a wide range of cancers, playing a role in tumor development and metastasis [22]. In the context of this study, annexin A3 is important in the immune response, acting as a signal for apoptotic cells to be consumed by nearby phagocytes, as well as being highly expressed in T and B lymphocytes [23]. In addition, an increase in ANXA3 transcripts in the blood of sepsis patients has been observed. The protein itself increases the proliferation of cancer cells and regulates neutrophil apoptosis [24]. Other studies clearly showed the important role of ANXA3 in protecting the myocardium from ischemic damage. Silencing this gene in rats most likely promotes heart muscle healing through activation of the PI3K/Akt signaling pathway [25]. Research has shown that in most species, including humans, fallopian tubes show an affinity for sperm. This indicates a possibly longer storage time for sperm in the female genital tract [26]. ANXA3 is also expressed in neutrophils, where its association with granules suggests a role in granule fusion, as well as neutrophil aggregation [9,27]. It was associated with the “defense response”, “cytokine secretion” and “cytokine production” ontological groups. The next gene, interferon-induced protein with tetratricopeptide repeats 1 (IFIT1), was upregulated 27.75-fold and encodes a protein that is important for innate immunity defense, specifically viral infection [28]. IFIT1 protects cells through recognition of specific RNA virus signatures and translational inhibition [29]. It was associated with the “defense response to other organism”, “defense response”, “defense response to virus” and “cytokine-mediated signaling pathway” ontological groups. In a 2016 study, IFIT1 was shown to be upregulated during the early antral follicle to small antral follicle transition in vivo, but was downregulated between early antral follicles and in vitro cultures of GC and oocyte complexes, highlighting it as an important determinant in the differences between in vivo and in vitro cultures’ gene expression profiles [30]. Furthermore, the next of the genes, major histocompatibility complex Class II, DP alpha 1 (HLA-DPA1), was upregulated 24.10-fold. It codes for a receptor protein that is vital for antigen-presenting cells, for example, in presentation of the hepatitis B virus to CD4+ helper T cells following infection [31]. It was associated with the “defense response”, “cytokine production” and “cytokine-mediated signaling pathway” ontological groups. In turn, myxovirus (influenza virus) resistance 1, interferon-inducible protein p78 (mouse) (MX1) was upregulated 19.60-fold during CC culture and was associated with the “defense response to other organism”, “defense response”, “defense response to virus” and “cytokine-mediated signaling pathway” GO BP terms. It codes for a GTPase protein, which aids in the antiviral response by targeting viral nucleocapsids [29]. The GTPase also positively correlates with follicular atresia and acts as a marker of oocyte quality, as demonstrated by a 2017 bovine-based study [32]. The next genes encode proteins of the keratin family. Keratin 8 (KRT8) encodes a Type II keratin which regularly forms intermediate filament structures with Type I keratin. Keratin 18 (KRT18) is most often found in the epithelial cytoplasm. These proteins have a functional role in cellular stability and structure, signal transduction and differentiation. KRT8 showed a 16.04-fold increase in expression, while KRT18 was upregulated 14.55-fold. These proteins were associated with the ontological group “cytokine-mediated signaling pathway”. Intermediate filaments Keratin 8 and 18 are known to prevent apoptosis in GCs by reducing the expression of the cell surface death receptor Fas [33]. The last upregulated gene, major histocompatibility complex Class II, DR alpha (HLA-DRA) is expressed in antigen-presenting cells such as macrophages and B lymphocytes. It was associated with the GO BP terms “cytokine-mediated signaling pathway” and “defense response”, and showed a 15.36-fold increase in expression throughout in vitro culture of human cumulus cells. 

Differential regulation of genes mapped to the “TNF signaling pathway” KEGG pathway was identified. All of these genes were upregulated after 7 days of human cumulus-oophorus cell culture in vitro. Cytokines were originally identified as the products of immune system cells, as a mediator of the immune response. However, as proteins, cytokines are involved in many processes related to the proper functioning of the body: they stimulate cell growth and regulate cell differentiation, and may also induce/modulate the expression of other cytokines. Moreover, research has indicated that these proteins are also produced by normal cells that are not related to the immune system. Their participation in the growth of the ovarian follicle and steroidogenesis, as well as in the recruitment and activation of leukocytes involved in ovulation and ovarian remodeling after ovulation has also been indicated [34,35]. One of the selected ontological groups suggests the participation of cytokines in the initiation of molecular signals through their binding of cell surface receptors and regulation of further cellular processes. 

Research has shown that TNF (tumor necrosis factor) and its specific receptors are present in the corpus luteum (CL) of many species of mammals, including humans. TNF plays a role not only in the proper formation of the CL but also throughout the female estrus cycle. TNF seems to play a luteolytic and luteotropic role in CLs, while the Fas ligand (one of the super-family members) is recognized as a factor of apoptosis [36]. In studies carried out in mice, both TNF alpha mRNA and the protein itself were observed in the oocytes of normal follicles with double or more layers of granular cells. However, in the oocytes of primary follicles, neither mRNA nor the protein itself was observed [37]. Moreover, it is known that the immunoreactivity activity is greater in older CLs than in those produced in the last estrus cycle. The presence of TNF-alpha has also been demonstrated in a conditioned medium after the cultivation of GCs [38] and in the follicular fluid [39]. It is believed to play a role in stimulating the proliferation of GCs and the production of progesterone during ovulation and the formation of the corpus luteum [39,40,41]. It has also been indicated that TNF may participate in the differentiation of the values of GCs [42] and influence their apoptosis [43]. In addition to TNF, IL-1β is another important cytokine involved in the control of follicle growth [44]. Human oocytes as well as the cumulus-oophorus express TNFR2 [45]. Ovarian TNF expression is most likely regulated by follicular development, ovulation and corpus luteum atrophy. In contrast, TNF secreted from the mature follicle may act as a paracrine factor inhibiting the development of smaller follicles; on the other hand, it stimulates the production of progesterone in the dominant follicle [46]. It has also been suggested that TNF-alpha is secreted by CCs and that the relevant receptors are located in oocytes. Thus, TNF most likely promotes the aging of oocytes [47]. Other studies have indicated that the overproduction of TNF-alpha may be associated with fertility disorders, as well as miscarriages or recurrent implantation failure syndrome [48]. It has been indicated TNF and LIF (leukemia inhibitory factor) are related to the development and implantation of embryos. Research on the level of LIF and TNF-alpha in spent culture medium has suggested that LIF plays a significant role in early embryogenesis and is a necessary factor for the proper implantation of the embryo. On the other hand, a high concentration of TNF was observed in the spent culture medium of embryos whose implantation was unsuccessful [49].

## 5. Conclusions

Almost every gene of interest involved in cytokine action and metabolism-mediated signaling pathways in immunological defense was highly upregulated throughout the in vitro culture of human cumulus cells. Of the 122 cytokine-associated genes, this research focused on the most upregulated genes. The results indicated a change in the properties of CCs under the conditions of primary culture in vitro. Changing the environment as well as changing the properties of CCs may be related to the effectiveness of the IVF procedure. There is a high level of predicted interaction between these genes, including upregulation of the focal adhesion and TNF signaling pathways. Further studies into the most differentially expressed genes and analyses of the gene interaction networks involved in immunological defense could improve our understanding of gene function in vivo throughout the lifespan of the oocyte. Understanding the molecular and cellular involvement of the immune system in follicular selection, maturation and ovulation is vital to continuing research across many areas involving the female reproductive system. Additionally, upregulation of the genes involved in immunological defense in cumulus cell cultures, and the similarities between ovulation and the inflammatory response remain to be investigated further.

## Figures and Tables

**Table 1:** Oligonucleotide. Sequences of primers used for RT-qPCR.

Gene	Primer Sequences (5′-3′)	Product Size (bp)
KRT18	CACAGTCTGCTGAGGTTGGA	164
	GAGCTGCTCCATCTGTAGGG	
HLA-DRA	CATGACAAAGCGCTCCAACT	231
	TGGAACTTGCGGAAAAGGTG	
KRT8	TGAGGTCAAGGCACAGTACG	161
	TGATGTTCCGGTTCATCTCA	
HLA-DPA1	CAAGAGCCAATCCAGATGCC	230
	ATGGAGTTTGTAGGGCAGCT	
MX1	CGGAATCTTGACGAAGCCTG	224
	CCTTTCCTTCCTCCAGCAGA	
IFIT1	GCGGTTTCCACATGACAACT	242
	ATTCATGAGG. GGCAGTCACA	
HPRT	TGGCGTCGTGATTAGTGATG	141
	ACATCTCGAGCAAGACGTTC	
ACTB	AAAGACCTGTACGCCAACAC	132
	CTCAGGAGGAGCAATGATCTTG	
GAPDH	TCAGCCGCATCTTCTTTTGC	90
	ACGACCAAATCCGTTGACTC	

**Table 2:** The seven most upregulated genes involved in cytokine action and metabolism-mediated signaling pathways activity during immunological defense processes in human cumulus-oophorus cells (CCs). Fold change refers to the Day 7–Day 1 comparison.

Gene Name	Gene Symbol	Fold Change	Adj. *p*. val.
Annexin A3	ANXA3	34.27	<0.05
Interferon-induced protein with tetratricopeptide repeats 1	IFIT1	27.75	<0.05
Major histocompatibility complex, class II, DP alpha 1	HLA-DPA1	24.10	<0.05
Myxovirus (influenza virus) resistance 1, interferon-inducible protein p78 (mouse)	MX1	19.60	<0.05
Keratin 8	KRT8	16.04	<0.05
Major histocompatibility complex, class II, DR alpha	HLA-DRA	15.36	<0.05
Keratin 18	KRT18	14.55	<0.05

## Data Availability

All the analyzed microarray data are available in the GEO database https://www.ncbi.nlm.nih.gov/geo/query/acc.cgi?acc=GSE149033.
